# If Stigmatized, Self-Esteem Is not Enough: Effects of Sexism, Self-Esteem and Social Identity on Leadership Aspiration

**DOI:** 10.5964/ejop.v12i4.984

**Published:** 2016-11-18

**Authors:** Angela Fedi, Chiara Rollero

**Affiliations:** a Department of Psychology, University of Turin, Turin, Italy; b Faculty of Psychology, University eCampus, Novedrate, Italy; NEOMA Business School, Reims, France

**Keywords:** ambivalent attitudes, leadership aspirations, self-esteem, social identification

## Abstract

Ambivalent sexism has many pernicious consequences. Since gender stereotypes also affect leadership roles, the present research investigated the effects of ambivalent sexism on envisioning oneself as a leader. Our studies tested the influence of sexist attitudes (toward women – Study 1 – and men – Study 2) on leadership aspiration, taking into account the interaction among ambivalent attitudes, personal characteristics (e.g. self-esteem), and group processes (e.g. level of identification with gender). Specifically, the current study used a 3 (sexism: hostile, benevolent, control) x 2 (social identification: high, low) x 2 (self-esteem: high, low) factorial design. 178 women participated in Study 1. Results showed that, although sexism was not recognised as a form of prejudice and did not trigger negative emotions, in sexist conditions high-identified women increase their leadership aspiration. In Study 2 men (*N* = 184) showed to recognise hostility as a form of prejudice, to experience more negative emotions, but to be not influenced in leadership aspiration. For both men and women self-esteem had a significant main effect on leadership aspiration.

## Introduction

Although women have gained access to almost sectors of work-life, substantial disparities in the career achievement of men and women persist, higher status jobs remaining predominantly a male prerogative (Eagly & Carli, 2004; Eagly & Karau, 2002). Gender disparity in leadership roles is neatly represented as the “leadership labyrinth” (Eagly, 2007; Eagly & Carli, 2007; Hoyt, 2010a, 2010b; Hoyt & Chemers, 2008), the unsanctioned barriers obstructing women from reaching higher status job positions.

Gender disparity in reaching leadership positions has been explained from different theoretical perspectives (e.g. Hoobler, Lemmon, & Wayne, 2014), taking into account personal, social, organizational and cultural factors. Some studies underlined the importance of leadership aspirations – conceived as the desire of and the possibility to perceive oneself in a power position (Boatwright & Egidio, 2003) – in influencing the tangible possibilities of career achievement. Women aspiring to leadership roles are target of negative stereotypes and prejudices which can impact their thoughts, feelings and emotions, behaviours and aspirations (Hoyt & Blascovich, 2007, 2010; Hoyt & Simon, 2011). Among these social and psychological barriers, ambivalent sexism plays a relevant role because of its apparently positive dimension resulting even more harmful than explicitly hostile attitudes (Barreto & Ellemers, 2005).

Classically, research deepened the detrimental effects of sexism on performance in a specific domain; only recently, scholars have highlighted the impact of gender stereotypes on self-perception and, in turn, on leadership aspirations (Simon & Hoyt, 2013). However, at best of our knowledge, no study has evaluated the effects of ambivalent sexism on the perception of oneself as a leader, together with the interaction among personal characteristics (e.g. self-esteem) and group processes (e.g. the level of identification with gender). The two studies here presented (involving both women – Study 1 – and men – Study 2) aimed at addressing this gap, investigating the influence of ambivalent sexism – along with the influence of self-esteem and social identification with gender – on leadership aspiration. In the following sections, we review leadership aspirations, the social and personal factors influencing them, and the gender gap in reaching leader job positions. Next, we draw from Ambivalent Sexism Theory, Social Identity Theory and Self-Categorization Theory to formulate the hypotheses of the two studies.

### Leadership Aspirations and Gender Stereotypes

Career aspiration refers to individuals’ desire to choose a specific occupation (e.g. Farmer, 1985; Gray & O’Brien, 2007; Nauta, Epperson, & Kahn, 1998). It can be considered a construct of three related items: job aspirations, job expectations, and educational aspirations (Creed, Tilbury, Buys, & Crawford, 2011). In parallel, leadership aspirations can be defined as the desire of and the possibility to perceive oneself in a power position. They are influenced by social, organizational and psychological factors (Boatwright & Egidio, 2003).

Many studies (for a review, see Watson & McMahon, 2005) underline the early impact of gender on the way pre-school and elementary school aged children think about their career options. Classically, the assessment of women’s career choice was based on the dichotomy between homemaking versus job orientation (Betz & Fitzgerald, 1987) and just in the 90’s scholars added the traditional versus non-traditional career choice and the prestigious versus non-prestigious job dichotomies (O’Brien & Fassinger, 1993). More specifically, in 1996, O’Brien defined career aspiration as “the degree to which women aspire to leadership positions and continued education within their careers” (as cited in Gray & O’Brien, 2007, p. 318), moving beyond traditional measures of employment choice.

Despite women have gained access to lower-level management positions, substantial differences in manager composition and difficulties for women in being recognised and accepted as legitimate leaders persist (Catalyst, 2012; Eagly & Carli, 2004; Eagly & Karau, 2002; Ridgeway, 2001). Moreover, research demonstrated that women do not aspire to leadership role as often as men (Rollero & Fedi, 2014; Skrla, Reyes, & Scheurich, 2000) or anticipate more problems than men when asked to envision themselves in powerful roles (Lips, 2001).

Leadership aspiration is recognised as one of the internal factors playing a role in determining the so-called “leaky pipeline” (Eagly & Carli, 2007; Shapiro et al., 2015), namely the diminishing number of women up in leadership position notwithstanding their better educational performances than men. Also the personal conflicts experienced by women and the internalization of values requested to aspire high position, poor self-image or self-esteem, socialized role expectations as well as modest career goals can deflate the possibility to reach powerful job positions (e.g. Fitzgerald, 2003; Gardiner, Enomoto, & Grogan, 2000; Wojtalik, Breckenridge, Gibson Hancox, & Sobehart, 2007).

Among the external barriers to career advancement for women, gender stereotypes have been hugely considered in the psychosocial literature. The link between stereotypes and gender gap in leadership can be analyzed from different perspectives (e.g. socialization and role development, Eagly & Karau, 2002; Shapiro et al., 2015; Wojtalik, Breckenridge, Gibson Hancox, & Sobehart, 2007; the development of gender-related social status, Ridgeway, 1997, 2001; Ridgeway & Balkwell, 1997; the gendered positioning, Katila & Eriksson, 2013). Particularly, since Lips’ (2000, 2001) research, several studies assessed the gender differences in vision of power and in possible selves (Markus & Nurius, 1986) linked to power roles. Recently, an interesting meta-analysis (Koenig, Eagly, Mitchell, & Ristikari, 2011) confirmed a strong tendency in considering leadership as culturally masculine.

With few exceptions (e.g. Schoon & Polek, 2011), results underline the higher difficult for women to imagine themselves as leaders: Even young women are less optimistic than men about holding leadership positions (e.g. Diekman, Goodfriend, & Goodwin, 2004; Hoyt & Simon, 2011; Killeen, López-Zafra, & Eagly, 2006; Lips, 2000, 2001). As Bosak and Sczesny (2008) demonstrated, this can occur because usually women associate traditional agentic (male) traits to leadership roles and view themselves as possessing less of these (supposed) required characteristics than men do. In sum, two stereotypes are at stake: one referring to the *masculine* leader; the other referring to the *communal* women (see Eagly & Karau, 2002).

### Ambivalent Attitudes Toward Women and Men

Literature about gender stereotypes has shown that stereotypes and prejudices do not need to be openly negative to be effective in their detrimental consequences. According to the Ambivalent Sexism Theory, three specific factors characterise structural relations between men and women in societies: a) patriarchy, as men are accorded more power than women; b) gender differentiation, as men and women are ascribed different traits and social roles; and c) sexual reproduction, that creates dependencies and intimacies between the sexes. These three factors together create both hostile and benevolent attitudes toward the other sex (Glick & Fiske, 1996, 2001). Specifically, Ambivalent Sexism Theory (Glick & Fiske, 1996, 2001) highlights the benevolent and hostile components of traditional attitudes towards both sexes. Referring to women, hostile sexism explicitly communicates antipathy, whereas benevolent sexism conveys an apparently positive portrait of women, although, similarly to hostile sexism, it relies on gender stereotypes and contributes to perpetuate gender inequalities. Concerning male gender, hostility toward men refers to overtly negative attitudes in response to sex power inequalities, while benevolence toward men represents positive or affectionate attitudes, based on recognising their dependence on women depicted as “pure” creatures who need male protection.

Research on sexism toward women has largely shown that the consequences of benevolent sexism can be even more pernicious than those exerted by explicitly hostile attitudes: Indeed, benevolent sexism is not clearly recognisable as a form of prejudice and thus it is more difficult to combat than hostile sexism (Barreto & Ellemers, 2005; Rollero & Fedi, 2012; Woodzicka & Ford, 2010). Specifically, many studies demonstrate that benevolent sexism leads to: legitimating domestic violence, sexual harassment and rape (Fiske & Glick, 1995; Sakall, 2001), decreasing self-esteem and performances (Dumont, Sarlet, & Dardenne, 2010; Rollero, 2015), maintaining of gender discrimination (Boasso, Covert, & Ruscher, 2012; Glick et al., 2000; Overall, Sibley, & Tan, 2011; Rollero, 2013).

Sexism toward men has been less studied. Similarly to benevolence toward women, benevolence toward men seems not to be recognised as prejudiced (Rollero & Fedi, 2012). Concerning the effects, Glick and Whitehead (2015) underlined that the endorsement of benevolence toward men predicts legitimacy of gender hierarchy, whereas hostility toward men is a significant predictor of perceived stability of the status quo.

Finally, few studies considered the impact of sexism on affective states. Among them, Barreto and Ellemers (2005) and Rollero and Fedi (2012, 2014) found that both men and women primed with ambivalent sexism experienced more anxiety and anger than those in the control condition. Although the judgemental process concerning the recognisability of sexism seems to be relatively independent from affective states, the salience of gender stereotypes can significantly foster negative emotions (Barreto & Ellemers, 2005).

### Self-Esteem, Social Identity and Gender Gap in Leadership

Sexism and its effects need multiple levels of comprehension. Indeed, if some personal characteristics (e.g. self-esteem) can be involved in the perception of and reactions to discriminatory attitudes, stereotypes are an intergroup phenomenon which also requires a collective frame (e.g. Social Identity Theory, Tajfel, 1978; Self-Categorization Theory, Turner, 1978).

Recently, Simon and Hoyt (2013; Hoyt & Simon, 2011) deeply explored the relation between gender stereotypes and leadership aspirations, underlining the impact of social comparison (with other female leaders or with women’s images from the media) on self-perception, and, in turn, on aspirations toward leadership roles of women. Among indicators of self-perception, self-esteem, namely the individual level of self-worth or self-acceptance across situations (Rosenberg, 1965), seems to play an important role in the relationship between people and power, either real or potential. A consistent amount of empirical results reports a positive association between self-esteem and leadership (e.g. Atwater, Dionne, Avolio, Camobreco, & Lau, 1999; Li, Arvey, & Song, 2011) and between self-esteem and leadership aspirations (Boatwright & Egidio, 2003). Shortly, Bass and Bass (2008) stated that leader’s self-esteem can bolster leader’s motivation and self-acceptance, close work relationships and independence. Moreover, whereas low self-esteem people are particularly sensitive to negative feedback, a high level of self-esteem can protect people from stress and negative experience or feedback (Velsor, Moxley, & Bunker, 2004). In a similar way, as hypothesized by stress-buffering theories (e.g. Brown & Harris, 1978), self-esteem can play a protective function against the pernicious effects of stereotypes and discrimination (Corning, 2002).

On the social side, Social Identity Theory and Self-Categorization Theory can be usefully applied to sexism and gender gap. The social identity approach to intra- and inter-group processes underlines the importance of the collective attributes of the group people belong to in order to define themselves in terms of social identity (Hogg & Abrams, 1988; Turner, Hogg, Oakes, Reicher, & Wetherell, 1987). More, Self-Categorization Theory (Turner et al., 1987) assumes that the extent to which a social category can be identity-defining depends on the salience of the category itself (Oakes, 1987): The social comparison with other categories of the social context is one of the factors strengthening the salience of a membership (Brown & Turner, 1981; Hoyt & Simon, 2011).

Historically – and currently – women belong to a low-status group and face stereotypes and discriminatory exclusion from power, resulting stigmatized (Hoyt & Simon, 2011; Major & O’Brien, 2005).

Negative stereotypes can provide the main framework to interpret the behaviour in a given domain. Consequently, stigmatized people can be treated or judged on the basis of the prejudiced attitudes, and they can experience the situational predicament termed “stereotype threat” that can deeply undermine their performance or aspiration (Davies, Spencer, & Steele, 2005). As hypothesized by the stereotype threat paradigm (Steele & Aronson, 1995; Steele, Spencer, & Aronson, 2002), not all people are equally susceptible to the effects of the stereotypes, but only individuals whose social identity is targeted by the stereotype are vulnerable to the stereotype threat. Talking about gender stereotype, only women should be affected by the negative effects of the priming (Davies et al., 2005). Nevertheless, according to Social Identity Theory (Tajfel & Turner, 1979), it is necessary to distinguish between the objective category membership and the subjective belongingness to that category: That is not all women experience the same sense of belonging to gender category. Consistently with the Social Identity Theory, women more vulnerable to the stereotype and to its negative effects are those who strongly identify themselves with their gender category.

As above described, many studies have investigated sex differences in envisioning oneself as a leader (e.g. Bosak & Sczesny, 2008; Davies et al., 2005; Hoyt & Simon, 2011; Killeen et al., 2006; Lips, 2000, 2001). However, at best of our knowledge, no study has examined the effects of ambivalent sexism on the perception of oneself as a leader, taking into account the interaction among sexist attitudes, personal characteristics (e.g. self-esteem), and group processes (e.g. the level of identification with gender).

### The Current Research

The two studies here presented aimed at investigating the influence of ambivalent sexism (toward women – Study 1, and toward men – Study 2) on leadership aspiration. The effect of sexism was tested along with the influence of self-esteem and social identification with gender. Before examining the effect on leadership, we tested: a) whether sexism was recognised as a form of prejudice; and b) the emotions participants experienced facing sexism.

In Study 1, since high-identifiers are likely to be more sensitive to stereotype threat, we expected that, when facing sexism, high-identified women would recognise both hostile and benevolent sexism as a form of prejudice (Hypothesis 1) and would experience more negative emotions (Hypothesis 2) than low-identified women. About the possibility of envisioning oneself as a leader, we hypothesized a main effect of self-esteem and an interaction among experimental condition, identification and self-esteem. Specifically, we suspected that – in the sexist conditions – high identification would make women more sensitive to stereotype (Hypothesis 3), but high self-esteem would protect them against such stereotypic beliefs (Hypothesis 4).

In Study 2, we expected that high-identified men would consider the sexist sources more prejudiced (Hypothesis 5) and would experience more negative emotions (Hypothesis 6) than low-identified men. However, since ambivalent attitudes toward men do not threaten their leadership abilities, we hypothesized that only self-esteem would play a significant role in envisioning oneself as a leader (Hypothesis 7).

## Study 1

### Method

#### Participants

A convenience sample of 178 women participated in the study. They were recruited via students’ assistance, according to the technique of convenience sampling. Their mean age was 42.95 years (*SD* = 11.05). The majority was high school graduated (46.63%) or had a lower level of education (31.46%), but there were also college graduated (21.91%). Most of the participants (84.27%) were still working, followed by housewives (10.11%), and a small percentage of retired people (2.80%) and unemployed people (2.80%).

#### Materials and Procedure

##### Independent variables

Participants completed a questionnaire. First, social identification and self-esteem were assessed. Three items measured their level of identification with the category of women: “I have lot in common with other women”, “I feel a strong sense of being connected to women”, “Being a woman is an important part of my self-image” (Cronbach’s alpha = .71) (Mael & Tetrick, 1992). Self-esteem was assessed with the Rosenberg Self-Esteem Scale (Rosenberg, 1965) (Cronbach’s alpha = .80).

##### Manipulation

Then participants were primed either with benevolent sexism (*N* = 61), hostile sexism (*N* = 62), or no sexism (control condition, *N* = 55). They read a description summarizing the results of a research concerning opinions about women in society. Following Barreto and Ellemers (2005), type of sexism (hostile vs benevolent) was manipulated in describing the results of such research, reporting statements based on Glick and Fiske’s (1996) subscales of Hostility or Benevolence toward women. In the control condition participants read that the research had demonstrated that women and men are similar.

Specifically, in the benevolent sexism condition women read the following story: “A recent study investigated the opinions about women in society. Participants agreed that many women have a quality of purity that few men possess and that women – compared to men – tend to have superior moral sensibility. Moreover, participants in the study agreed that no matter how accomplished he is, a man is not truly complete as a person unless he has the love of a woman, that women should be cherished and protected by men, and that men should be willing to sacrifice themselves in order to provide financially for the women in their lives”.

In the hostile sexism condition women read the following story: “A recent study investigated the opinions about women in society. Participants agreed that women are too easily offended and interpret innocent remarks as sexist, and that women seek to gain power by getting control over men. Moreover, participants in the study agreed that women exaggerate problems they get at work, that when women lose to men in a fair competition they typically complain being discriminated, and that most women do not appreciate fully what men do for them”.

In the control condition, women read the following story: “A recent study investigated the opinions about contemporary society. Participants in the study agreed that both men and women exaggerate problems they get at work, are too easily offended and seek to gain power by getting control over others. Moreover, participants in the study agreed that both men and women tend to have the same moral sensibility and are not truly complete as persons unless they have the love of a partner”.

Participants were randomly assigned to one of the three groups (hostile sexism, benevolent sexism, control condition). No significant difference emerged among the groups about age (*F* = 1.13, n.s.) and educational level (*F* = 1.84, n.s.).

*Dependent Variables.* Following previous studies (Barreto & Ellemers, 2005; Rollero & Fedi, 2012), participants were asked to what extent they thought that people who held the above opinions were prejudiced against women (perception of sexism) and to what extent they experienced different negative emotions (anger, indignation, irritation, disappointment, and frustration). Finally, participants rated the possibility of being a chief executive officer (CEO) of a company, regardless of their educational level and profession. All dependent measures were scored on 7-point rating scales ranging from (1) “not at all” to (7) “very much”.

### Results

Hypothesis testing for the current study used a 3 (sexism: hostile, benevolent, control) x 2 (social identification: high, low) x 2 (self-esteem: high, low) factorial design.

In the first analysis of variance (ANOVA) the perception of sexism was the dependent variable: No independent variable had a significant effect.

In the following ANOVAs, we tested the effects of the independent variables on each negative emotion. Only in the case of disappointment type of sexism was significant, *F*(2, 162) = 3.89, *p* < .05. In the hostile condition, women were more disappointed (*M* = 3.09, *SD* = 2.27) than in the benevolence (*M* = 2.12, *SD* = 1.73) and in the control (*M* = 2.20, *SD* = 1.68) conditions.

**Figure 1:**
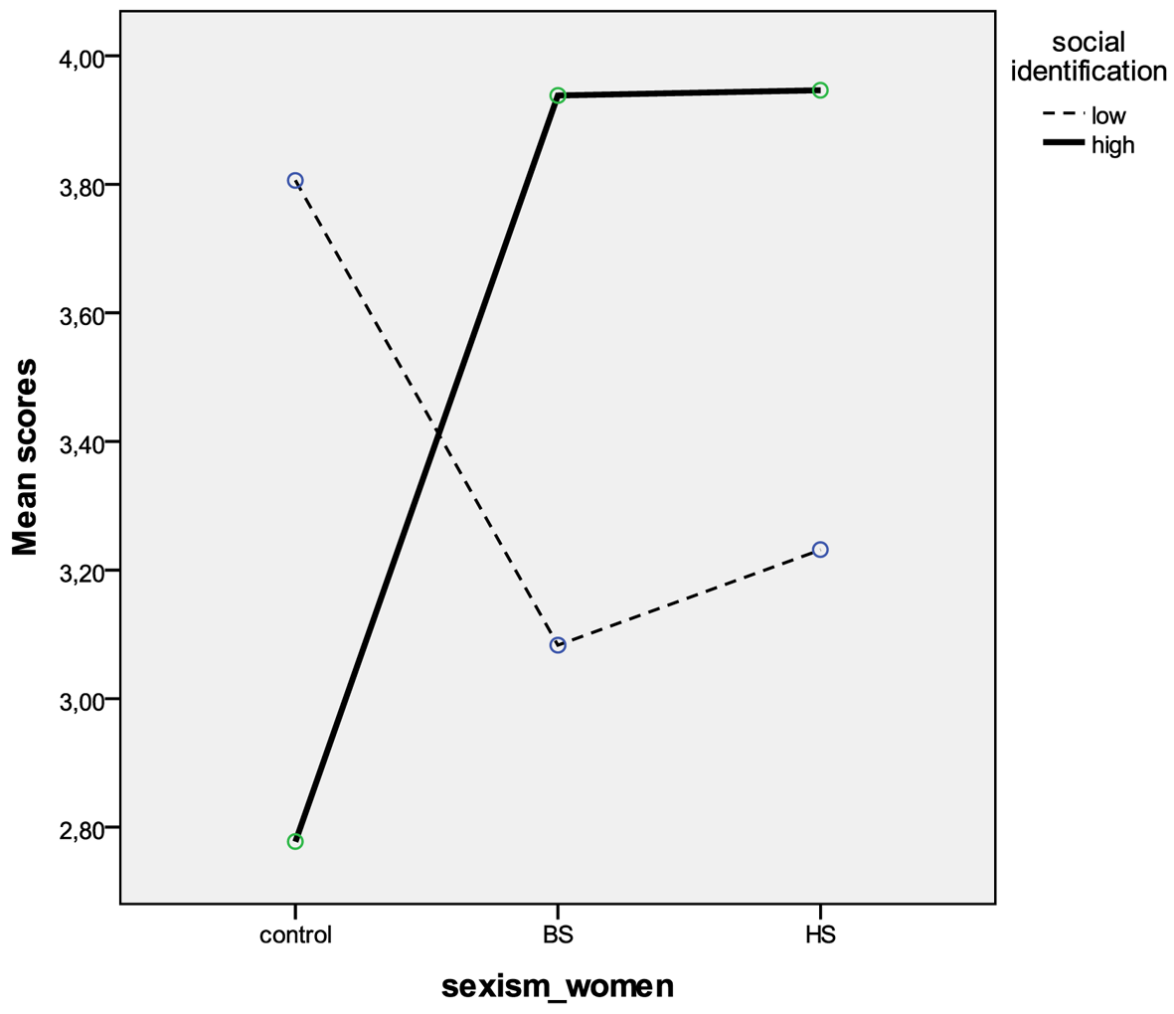
Interaction between experimental conditions and identification on women’s perception of being potential leaders.

Finally, the perception of being potential leaders was assessed. This ANOVA revealed a main effect of self-esteem, *F*(1, 161) = 4.50, *p* < .05, and a significant interaction between type of sexism and social identification, *F*(2, 161) = 3.46, *p* < .05. Women with high self-esteem were more likely to perceive themselves as potential leaders (*M* = 3.86, *SD* = 2.14) than women with low self-esteem (*M* = 2.97, *SD* = 2.19). High-identified women were more confident about the possibility of being a leader in both the sexist conditions than in the control condition, *F*(2, 91) = 2.91, *p* < .05, whereas low-identifiers showed similar scores across all the experimental conditions, *F*(2, 80) = 1.08, n.s. (Figure 1).

### Discussion

Both benevolent and hostile sexism were not recognised as a form of prejudice, as women report similar scores across the experimental and the control conditions. Moreover, sexism did not trigger negative emotions, with the only exception of disappointment. This last was more experienced by women who faced hostility. Contrary to our Hypotheses 1 and 2, identification had no influence.

Self-esteem, as expected, increases women’s leadership aspiration (Hypothesis 4). Also type of sexism played a key role, along with identification. Both hostility and benevolence do not change the self-perception of low identifiers, but they do foster the perception of high identified women as leader. Therefore, prejudice seems not to threaten all the members of the targeted category, as only female participants for whom gender identity is more relevant are vulnerable to stereotype threat. However, the reaction to stereotype threat was in part unexpected (Hypothesis 3): Regardless their levels of self-esteem, high-identified participants increase their leadership aspiration, so that gender identification seems to be a key factor in fostering reactance to stereotype threat. In gender studies, the importance of reactance has been discovered about performance (e.g. Dardenne, Dumont, & Bollier, 2007; Kray, Thompson, & Galinsky, 2001) and self-perception (Rollero & Fedi, 2012). Present findings seem to reveal a sort of unawareness of the reactance effect, as prejudices are not recognised neither foster negative emotions.

In general, findings about women are only partially in line with previous studies. This is due to the different variables considered: indeed, research has investigated either sex differences in envisioning oneself as a leader (e.g. Bosak & Sczesny, 2008; Davies et al., 2005; Hoyt & Simon, 2011; Killeen et al., 2006; Lips, 2000, 2001) or the recognisability of ambivalent sexism (Barreto & Ellemers, 2005; Rollero & Fedi, 2012). The effort made in the present study was instead assessing the effects of ambivalent sexism on the perception of oneself as a leader, taking into account the interaction among sexist attitudes, personal characteristics (e.g. self-esteem), and group processes (e.g. the level of identification with gender).

## Study 2

### Method

#### Participants

A total of 184 men were enrolled into the study. The average age was 40.58 years (*SD* = 10.99). About the education, the majority was high school graduated (46.84%), the 36.96% had a lower level of education, and the remaining (16.20%) were college graduates. Most of the participants were workers (94.65%), followed by retired people (3.80%) and a small percentage of unemployed people (1.55%).

#### Materials and Procedure

##### Independent variables

Participants completed a questionnaire very similar to that reported in Study 1. The identification with the category of men (Cronbach’s alpha = .69) and self-esteem (Cronbach’s alpha = .81) were assessed.

##### Manipulation

Then participants were primed either with benevolence toward men (*N* = 61), hostility toward men (*N* = 60) or no sexism (control condition, *N* = 63). They read a description summarizing the results of a research concerning opinions about men in society. Type of attitude (hostile vs benevolent) was manipulated in describing the results of such research, reporting statements based on Glick and Fiske’s (1999) subscales of Hostility or Benevolence toward men. In the control condition participants read that the research had demonstrated that women and men are similar.

Specifically, in the benevolence toward men condition men read the following story: “A recent study investigated the opinions about men in society. Participants agreed that, in emergencies, men are less likely to fall apart than women are, and that men are more willing to put themselves in danger to protect others. Moreover, participants in the study agreed that men are mainly useful to provide financial security for women”.

In the hostility toward men condition men read the following story:

“A recent study investigated the opinions about men in society. Participants agreed that even men who claim to be sensitive to women’s rights really want a traditional relationship at home, with a woman performing most of the housekeeping and child care. Moreover, participants in the study agreed that men usually try to dominate conversations when talking to women and that they will always fight to have greater control in society than women”.

In the control condition men read the following story:

“A recent study investigated the opinions about contemporary society. Participants in the study agreed that both men and women usually try to dominate conversations when talking to others and that they will always fight to have greater control in society than others. Moreover, participants in the study agreed that both men and women are willing to put themselves in danger to protect others and are mainly useful to provide financial security for their partner”.

They were randomly assigned to one of the three groups (hostility toward men, benevolence toward men, control condition). No significant difference emerged among the groups about age (*F* = 1.09, n.s.) and educational level (*F* = 1.51, n.s.).

##### Dependent variables

As in Study 1, perception of sexism and experience of negative emotions were assessed first. Then participants rated the possibility of being a chief executive officer (CEO) of a company, regardless of their educational level and profession. All dependent measures were scored on 7-point rating scales ranging from (1) “not at all” to (7) “very much”.

### Results

Hypothesis testing for the current study used a 3 (attitude toward men: hostile, benevolent, control) x 2 (social identification: high, low) x 2 (self-esteem: high, low) factorial design.

In the first ANOVA the perception of sexism was the dependent variable and type of attitude had a main effect, *F*(2, 172) = 5.66, *p* < .01: Participants considered the benevolent source less prejudiced (*M* = 3.05, *SD* = 2.10) than the hostile (*M* = 4.46, *SD* = 1.67) and the control (*M* = 4.05, *SD* = 2.11) ones.

In the following ANOVAs we tested the effects of the independent variables on each negative emotion. The main effect of type of attitude was significant for all emotions. In respect to benevolence, hostility toward men increased the experience of anger, indignation, irritation, disappointment, and frustration (Table 1).

**Table 1:** Sexist Attitudes Toward Men and the Experience of Negative Emotions

Emotion	*M* (*SD*)			*F*	Post hoc (Bonferroni)
	Hostility (H)	Benevolence (B)	Control (C)		
Anger	2.34 (1.83)	1.46 (0.86)	1.92 (1.48)	4.73*	H-B**
Indignation	2.50 (1.89)	1.54 (0.93)	1.96 (1.33)	5.62*	H-B**
Irritation	2.48 (1.87)	1.56 (1.05)	2.21 (1.63)	4.55*	H-B**
Disappointment	2.95 (1.97)	1.93 (1.44)	2.39 (1.72)	4.20*	H-B**
Frustration	2.21 (1.84)	1.36 (0.76)	1.80 (1.51)	4.37*	H-B**

*p < .05. **p < .01.

Finally, the perception of being potential leaders was assessed. This ANOVA revealed a main effect of self-esteem, *F*(1, 171) = 7.70, *p* < .01. Men with high self-esteem were more likely to perceive themselves as potential leaders (*M* = 4.50, *SD* = 2.27) than those with low self-esteem (*M* = 3.46, *SD* = 2.01). Attitude toward men played no significant role on this dependent variable.

### Discussion

If hostility toward men is recognised as prejudiced, benevolence not only is not recognised as prejudiced, but it is considered less sexist than the control condition. Indeed, facing the control condition (i.e. men and women described as similar), men’s perception of sexism is analogous to that reported in the hostility condition. Only benevolence seems to men not to be prejudiced. Attitudes toward men also have an impact on each investigated emotion. In respect to benevolence, when men face hostility they become angrier, more indignant, irritated, disappointed, and frustrated. Contrary to expectations (Hypothesis 5 and 6), identification plays no role on recognisability and emotions. These findings may be connected to the content of sexist prejudice, as benevolence toward men expresses a positive evaluation of the power differentiation between genders (Glick & Fiske, 1999). In this sense, men may perceive as positive attitudes underlying their right to dominance and power.

However, although men recognise hostility as a form of prejudice, and although they experience more negative emotions, they are not influenced when they have to think about holding a leadership position. Thus, sexism has an impact on emotional responses, but not on the perception of being potential leaders (Hypothesis 7). Again, this might depend on the contents of prejudiced attitudes toward men since both attitudes foster a representation of men as well-suited for leadership positions, but since hostility is negative in tone, it enhances negative emotions and seems more prejudiced.

## General Discussion

Our research aimed at investigating the influence of ambivalent sexism on the possibility of envisioning oneself as a leader. The effect of sexism was tested along with the influence of individual dimensions (e.g. self-esteem) and social processes (e.g. identification with gender). Moreover, before testing the effects on leadership, we have investigated whether both men and women recognise sexist attitudes and which emotions they experience.

Concerning recognisability of sexism, findings show different results between men and women. Indeed, women do not recognise either benevolent or hostile attitudes as prejudiced, reporting similar scores across the experimental and the control conditions. Men, on the contrary, define the hostile and the control conditions as more sexist than the benevolent one. In line with these results, women dealing with sexism have almost no negative emotional reaction (they only feel disappointed in the hostility condition), whereas men facing hostility become angrier, more indignant, irritated, disappointed, and frustrated.

When leadership becomes a salient topic, men and women show again different patterns. Men’s envisioning is affected only by self-esteem. This last is significant also for women, consistently with studies about the positive association between self-esteem and leadership aspirations (Boatwright & Egidio, 2003).

However, in the female sample, both sexism and identification play a key role. Indeed, hostility and benevolence foster the perception of high identified women as potential leaders. In other words, although high identified women do not recognise sexist attitudes as a form of prejudice and do not experience significant emotional reactions, they perceive the stereotype threat when a counter-stereotypical role, i.e. leader, becomes salient. On the contrary, men do consider hostility as a form of sexism, do report negative emotions, but do not feel threatened by sexist attitudes when leadership is relevant.

This might depend on the content of sexism toward both genders. Both men and women are targeted by some sexist attitudes (Glick & Fiske, 1996, 1999). However, since sexism is based to the endorsement of traditional gender roles, attitudes toward women discourages those who aspire at holding leadership positions, whereas attitudes toward men emphasize their power. Indeed, if benevolence expresses a positive evaluation of the power differentiation between men and women, hostility, although negatively toned, presumes that men will always have a power advantage (Glick et al., 2004).

In line with other theoretical and empirical works (e.g. Brewer & Kramer, 1985; Costarelli & Callà, 2007; Turner, 1978), our findings highlight the importance of the level of in-group identification for the meaning people give to an intergroup situation and the need for high-identified ones to maintain a more positive view of their group.

Indeed, high social identification with the gender category seems to have a “protective” role for women, allowing them to envision the possibility of a power position despite the sexist situation. Since high identifiers are more sensitive to the stereotype threat, we can consider their enhanced possibility of becoming a CEO as a sort of “reactance”. Reactance Theory (Brehm, 1966; Brehm & Weinraub, 1977) states that when a restriction is seen as unfair, people can get an unpleasant feeling that can play as an intense motivational state to get around the restriction. As above discussed, the importance of reactance has been investigated about performance (e.g. Dardenne, Dumont, & Bollier, 2007; Kray, Thompson, & Galinsky, 2001) and self-perception (Rollero & Fedi, 2012). If this perspective can satisfactory held here, we must underline the unaware – both cognitive and emotional – nature of the reactance effect, since the prejudiced beliefs are not explicitly recognised neither arouse emotional reactions *per se*. However, the study confirms the importance of approaching stereotypes in a group perspective: Social processes are particularly relevant when involving groups targeted with negative stereotypes and when inequalities embedded in the status quo are at stake.

Of course, the present study shows some limitations, as some central constructs could be operationalized in multiple ways. Since literature is focusing on different kinds of leadership roles and styles (e.g. Eagly & Johannesen-Schmidt, 2001; Eagly, Johannesen-Schmidt, & van Engen, 2003; Killeen et al., 2006), further inquiries should deeper investigate the influence exerted by ambivalent attitudes on the possibility of holding other power positions (e.g. scientific or political leadership). Similarly, self-esteem could be considered as a global measure or as a domain-specific self-evaluation, for example in terms of leadership effectiveness (Chemers, Watson, & May, 2000).

Despite this, the present research bears implications for theoretical issues both on effects of ambivalent attitudes and on gender gap in leadership, even representing one of the rare studies on adult sample. Moreover, since the effects of ambivalent attitudes on men are largely ignored in the literature, the choice to examine both women’s and men’s leadership aspiration could be a promising field of study.
